# Neuroradiological differentiation of white matter lesions in patients with multiple sclerosis and Fabry disease

**DOI:** 10.1186/s13023-022-02187-y

**Published:** 2022-02-05

**Authors:** Jakob Rath, Olivia Foesleitner, Lukas Haider, Hubert Bickel, Fritz Leutmezer, Stephan Polanec, Michael A. Arnoldner, Gere Sunder-Plassmann, Daniela Prayer, Thomas Berger, Paulus Rommer, Gregor Kasprian

**Affiliations:** 1 Department of Neurology, Medical University of Vienna, Währinger Gürtel 18-20, 1090 Vienna, Austria; 2 Department of Biomedical Imaging and Image-Guided Therapy, Medical University of Vienna, Vienna, Austria; 3 Division of Nephrology and Dialysis, Department of Medicine III, Medical University of Vienna, Vienna, Austria; 4 NMR Research Unit, Queens Square MS Centre, Department of Neuroinflammation, UCL Queen Square Institute of Neurology, Faculty of Brain Science, University College London, Russel Square, London, UK

**Keywords:** Fabry disease, Multiple sclerosis, Magnetic resonance imaging, Interrater reliability, Diagnosis

## Abstract

**Objective:**

White matter lesions (WML) in multiple sclerosis (MS) differ from vascular WML caused by Fabry disease (FD). However, in atypical cases the discrimination can be difficult and may vary between individual raters. The aim of this study was to evaluate interrater reliability of WML differentiation between MS and FD patients.

**Materials and methods:**

Brain MRI scans of 21 patients with genetically confirmed FD were compared to 21 matched patients with MS. Pseudonymized axial FLAIR sequences were assessed by 6 blinded raters and attributed to either the MS or the FD group to investigate interrater reliability. Additionally, localization of WML was compared between the two groups.

**Results:**

The median age of patients was 46 years (IQR 35–58). Interrater reliability was moderate with a Fleiss' Kappa of 0.45 (95%CI 0.3–0.59). Overall, 85% of all ratings in the MS group and 75% in the FD group were correct. However, only 38% of patients with MS and 33% of patients with FD were correctly identified by all 6 raters. WML involving the corpus callosum (*p* < 0.001) as well as juxtacortical (*p* < 0.001) and infratentorial lesions (*p* = 0.03) were more frequently observed in MS patients.

**Conclusion:**

Interrater reliability regarding visual differentiation of WML in MS from vascular WML in FD on standard axial FLAIR images alone is only moderate, despite the distinctive features of lesions in each group.

## Background

White matter lesions (WML) are a frequent neuroradiological finding in brain MRI with a large number of underlying causes [1]. Two of the most common etiologies are multiple sclerosis (MS) and vascular disorders causing small vessel disease (SVD), each with distinct and characteristic features [2, 3].

Neuroimaging criteria for the diagnosis of MS focus on the number and topography of lesions and their characteristic dissemination in space and time [2, 4, 5]. MS lesions exhibit characteristic features such as ovoid shape, typically periventricular along the deep medullary veins perpendicular to the lateral ventricles or involving juxtacortical U-fibers, which facilitates differentiation from other disorders with WML [3]. In contrast, vascular lesion due to SVD typically do not involve the U-fibers, differ in shape [6, 7] and are commonly associated with lacunae or microbleeds. While vascular WML are frequently found in the elderly population [8], etiologies in the young include rare genetic disorders such as Fabry disease (FD; MIM 301500), an X-linked lysosomal storage disease [9]. Cerebrovascular involvement in FD can lead to stroke and subcortical WML, the latter caused by microangiopathy due to endothelial deposition of glycosphingolipids. WML are found in a majority of patients as singular, multiple or confluent T2-weighted hyperintense MRI lesions [10]. However, a recent study showed that MRI findings alone could not differentiate patients with Fabry disease from other vascular patients. [11].

In the majority of patients, the clinical and neuroradiological differentiation of MS from its mimics is generally straightforward. Yet in atypical cases the classification of WML can be challenging, especially at singular routine MRI studies, when clinical information is limited or when progression of vascular WML mimics MS’s typical dissemination in time [3, 12, 13]. Moreover, radiological MS criteria are also met by a proportion of patients with WML due to other disorders [14–16] and an initial misdiagnosis as MS has been described occasionally in FD patients [17–20]. Furthermore, the interrater reliability regarding the discrimination of WML due to MS from those in SVD is not fully known and likely depends on the expertise and experience of the rater [21].

As misdiagnosis of patients with WML may lead to wrong and potential harmful treatments, the aim of this study was to examine the interrater reliability of WML discrimination between matched patients with MS and FD based on standard MRI axial FLAIR images. Additionally, we investigated the spatial WML distribution between the two groups. The advantage of WML evaluation in FD is the younger age at which WML occur facilitating comparison with age-matched MS patients. Furthermore, FD is a genetically confirmed disorder and the etiology of the WML can be consequently assumed to be vascular in this cohort.

## Methods

### Patients

Brain MRI data of 21 patients with a genetically confirmed diagnosis of FD who had WML and 21 patients with confirmed MS according to the McDonald diagnostic criteria [4] were retrospectively analyzed. Ethical approval was obtained from the Ethics Committee of the Medical University of Vienna and the requirement to obtain patient consent was waived for this retrospective study (EC-Numbers: 1135/2015 and 1464/2017). The study was carried out in accordance with the World Medical Association Declaration of Helsinki and relevant local regulations. Patients were matched based on their sex, age and lesion load by one author (O.F.). Lesion load was visually assessed and a semiquantitative classification into singular lesions, beginning confluence and large confluent areas was performed. The classification was based on the commonly used Fazekas scale for vascular lesions in the periventricular and deep white matter [22] but extended to WML in general regardless of location to allow assessment in MS patients as well.

### Imaging analysis

MRI scans were performed between 2009 and 2017 at the Division of Neuro- and Musculoskeletal Radiology of the Department of Biomedical Imaging and Image-guided Therapy of the Medical University of Vienna. All sequences were acquired at 3 Tesla (FD group: T2-FLAIR: matrix: 256 × 256, slice thickness 3 mm, TE 126 ms, TR 10,000 ms, TI 2500 ms; MS group: T2-FLAIR 3D: matrix 512 × 512, TE 400 ms, TR 6000 ms, TI 2100 ms, 1 × 1x1 mm voxel size) on a Siemens TIM trio scanner except for 2 FD patients who were measured at a Philips Medical System Ingenia scanner. Additionally, T1-weighted sequences were evaluated in all patients. Contrast-enhanced T1-weighted sequences were available in 14 FD patients and 20 MS patients.

#### Interrater reliability

Interrater reliability of WML classification was assessed by randomized evaluation of pseudonymized axial FLAIR images of all 42 patients by 6 raters who had no clinical information and no access to other MR sequences. The raters consisted of 2 neuroradiologists, 2 general radiologists and 2 neurologists specialized in MS. Raters were asked to assign each patient to either the MS or the FD group based on visual assessment of WML on axial FLAIR images.

#### Secondary imaging analyses

To investigate whether the MS and FD groups differed as expected regarding the distribution of WML, FLAIR- as well as contrast-enhanced T1w-sequences were analyzed by one author (J.R.) without blinding, who did not participate in the interrater analysis, with regard to the presence of:

- Corpus callosum involvement of WML (either callosal lesions or paracallosal lesions extending into the corpus callosum),
- Contrast-enhancing lesions,
- ≥ 1 infratentorial WML and
- ≥ 1 juxtacortical WML (defined as clearly involving the subcortical U-fibers).

Additionally, T1w-sequences were analyzed by the same author with regard to:

- Lacunar lesions (< 15 mm) or evidence of prior territorial or embolic stroke and
- A pulvinar sign (hyperintensity of the pulvinar on T1w-images).

### Statistical analysis

SPSS 26 software package (IBM Corp. Released 2019. IBM SPSS Statistics for Macintosh, Version 26.0. Armonk, NY: IBM Corp), R version 4.02 (R Core Team, 2020. R: A language and environment for statistical computing. R Foundation for Statistical Computing, Vienna, Austria) and R Studio version 1.3.959 (RStudio Team, 2020. RStudio: Integrated Development for R. RStudio, PBC, Boston, MA) were used for statistical analysis.

Categorical data was compared using the Chi-square test or Fisher exact test and the Mann–Whitney U or Student’s t-test was used for comparison of continuous variables, as applicable. Interrater reliability measures included Fleiss' Kappa for multiple raters and Krippendorff's alpha with bootstrap confidence intervals [23]. Interrater reliability measures were calculated for all patients and all 6 raters. To check for outliers dropping of one rater was performed for all raters. Interpretation of Fleiss’ Kappa was based on the suggested guidelines by Altman: < 0.20 = poor, 0.21–0.40 = fair, 0.41–0.60 = moderate, 0.61–0.80 = good, 0.81–1.00 = very good [24].

Additionally, the descriptive number of correct ratings per patient and rater was analyzed and characteristics of patients with perfect agreement (i.e., all 6 raters correctly assigned the patient to the MS or FD group) were compared to patients who had divergent ratings using multivariate logistic regression analysis with sex, group and age and each of their interactions as covariates. For the secondary analyses at the group level performance measures were calculated. *P* ≤ 0.05 was considered statistically significant. For the secondary imaging analyses, *p* values were adjusted to account for multiple comparisons using the Holm-Bonferroni sequential correction method [25].

## Results

Table 1 shows baseline characteristics of the 21 MS and 21 FD patients. Groups were well matched regarding age, sex and lesion load. As expected, cardiomyopathy and chronic kidney disease were more frequently observed in the Fabry disease group. The MS group included patients with relapsing–remitting as well as secondary and primary progressive disease courses with a median EDSS of 3.5 points.

**Table 1:** Baseline characteristics

	Fabry disease n = 21	Multiple sclerosis n = 21	*p* values
Sex			0.76
Female	12 (57%)	13 (62%)	
Male	9 (43%)	8 (38%)	
Age median (IQR, range)	46 (27; 19–67)	45 (25; 19–61)	0.80
TIA or Stroke	2 (8.7%)	0	0.49
Cardiomyopathy	13 (65%)	0	< 0.001^‡^
Chronic kidney disease	13 (65%)	0	< 0.001^‡^
Enzyme replacement therapy	16 (76%)	NA	
Lesion load			0.27
Singular lesions	17 (81%)	14 (67%)	
Beginning confluence	2 (10%)	6 (28%)	
Large confluent areas	2 (10%)	1 (5%)	
MS type			
RRMS	NA	13 (62%)	
SPMS	NA	7 (33%)	
PPMS	NA	1 (5%)	
EDSS median (IQR; range)	NA	3.5 (3.3; 0–7)	
CSF (pos. OCBs and/or intrathecal IG production)	NA	20 (95%)	

Clinical features at the time of brain MR imaging. Lesion load was visually assessed and rating was based on the classification of Fazekas et al. [22] for white matter lesions but extended to all lesion locations. *CSF* cerebrospinal fluid, *EDSS* expanded disability status scale, *IG* immunoglobulin, *IQR* interquartile range, *OCB* oligoclonal bands, *TIA* transient ischemic attack, *RRMS* relapsing–remitting multiple sclerosis, *SPMS* secondary-progressive multiple sclerosis, *PPMS* primary-progressive multiple sclerosis

^‡^Statistically significant

### Interrater reliability

Fleiss' Kappa was 0.45 (95%CI 0.3–0.59) for all ratings of the 42 patients by the 6 raters and Krippendorff's alpha was also 0.45 (95%CI 0.3–0.6). Dropping one of the raters from the analysis resulted in Fleiss' Kappa values between 0.4 and 0.54.

80% (202/252) of all ratings were correct with 85% (107/126) and 75% (95/126) in the MS and FD group, respectively. In the MS group, correct ratings did not differ between relapsing–remitting (84.6%; n = 13) and secondary or primary progressive (85.4%; n = 8) disease courses. Correct ratings in all patients (FD and MS) were slightly higher in the 20 patients below the median age of 46 years compared to the 22 patients at or above the median age with 85% and 76%, respectively.

**Fig. 1:**
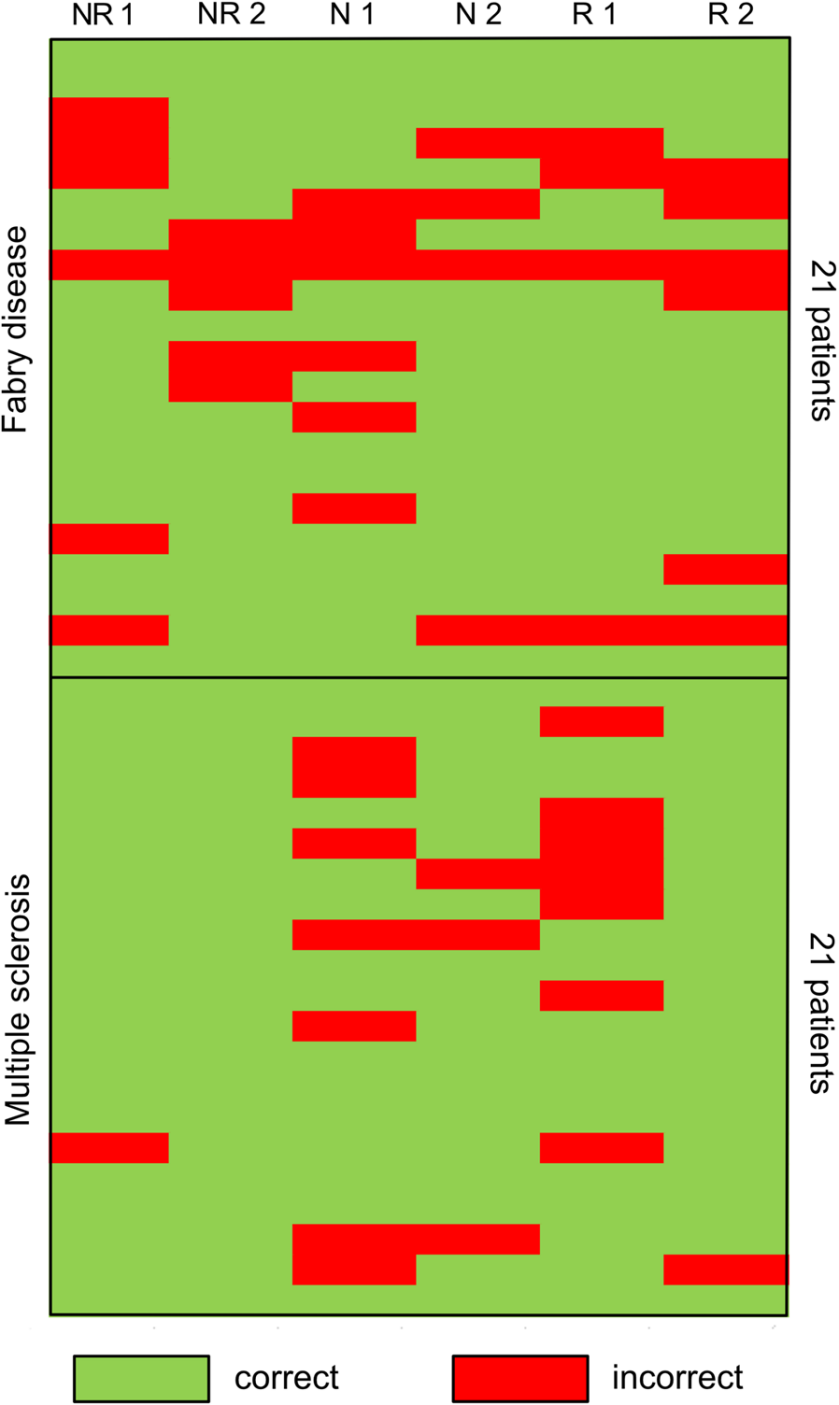
Individual ratings of all patients. The classification was based on pseudonymized FLAIR images of each of the 42 patients by 6 raters (each column represents one rater with NR 1 denoting neuroradiologist 1, NR 2 neuroradiologist 2, N 1 neurologist 1, N 2 neurologist 2, R1 general radiologist 1 and R 2 general radiologist 2). The upper half shows ratings for the 21 FD patients (each row of squares represents one patient with green squares representing correct ratings and red squares incorrect ratings) and the lower half ratings of 21 matched MS patients

**Fig. 2:**
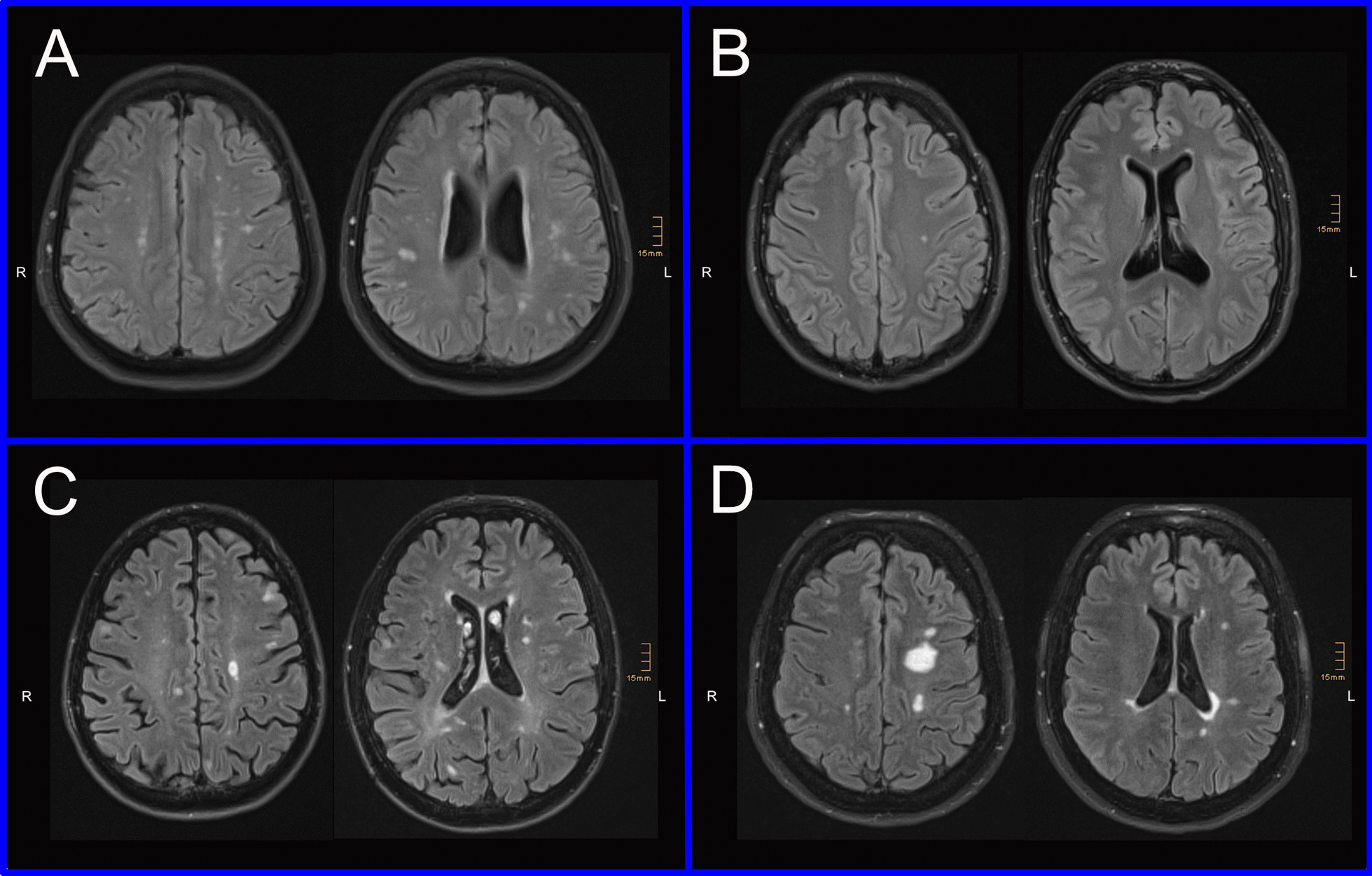
Examples of white matter lesions on FLAIR images. **a** 65-year-old woman with Fabry disease receiving enzyme replacement therapy; all raters incorrectly classified images as MS. **b** 31-year-old male patient with Fabry disease receiving enzyme replacement therapy; 5 of 6 raters correctly classified the images as FD. **c** 36-year-old male patient with relapsing remitting multiple sclerosis (EDSS 3.5, therapy with alemtuzumab at the time of MR scan; 4 of 6 raters correctly classified the images as MS. **d** 61-year-old female patient with secondary progressive multiple sclerosis (EDSS 6.5, no therapy at the time of MR scan; 5 of 6 raters correctly classified the images as MS

The range across individual raters for a correct diagnosis was 66.7–100% in MS patients and 71.4%-82% in FD patients. Regarding training background, the numerical rates of a correct MS classification varied between neuroradiologists (95% and 100%), neurologists (67% and 86%) and general radiologists (95% and 67%). The ratings for FD did not differ substantially (71%, 76%, 71%, 81%, 81% and 71%, respectively). However, only 33% (7/21) of FD patients and 38% (8/21) of MS patients were correctly rated by all 6 raters. Patients who had ratings with perfect agreement did not differ from patients with divergent ratings regarding their age (*p* = 0.10), sex (*p* = 0.61), or group (MS vs. FD; *p* = 0.76). Figure 1 shows the individual classification of each rater per patient and Fig. 2 examples of FLAIR images of patients with MS and FD.

### Secondary imaging analyses

Table 2 shows group comparisons of MRI data. MS patients had a significantly more frequent involvement of the corpus callosum, the juxtacortical region and infratentorial regions. Lacunar lesions were numerically more frequent in the FD group but differences were not statistically significant with adjusted p-values. Contrast-enhancing lesions did not differ in a statistically significant way; however, no occurrence was observed in the FD group. A pulvinar sign was not present in any of our FD patients. Measures of diagnostic performance for WML localization and lacunar lesions are shown in Table 3. A high specificity was found for WML in the corpus callosum (95%), juxtacortical lesions (86%) and infratentorial lesions (86%) for MS vs. FD patients and for lacunar lesions (95%) for FD vs. MS patients. Sensitivity for the discrimination of MS from FD was high for corpus callosum involvement and juxtacortical lesions (81%) but only low to moderate for infratentorial WML (52%) and lacunar lesions (FD vs. MS; 33%), respectively.

**Table 2:** Results of secondary imaging analyses

	Fabry disease (n = 21)	Multiple sclerosis (n = 21)	Adjusted *p* values
Corpus callosum involvement	1 (5%)	17 (81%)	< 0.001^‡^
Juxtacortical lesions	3 (14%)	17 (81%)	< 0.001^‡^
Infratentorial lesions	3 (14%)	11 (52%)	0.03^‡^
Contrast enhancing lesions*	0 /14	2/20 (10%)	0.50
Lacunar lesions	7 (33%)	1 (5%)	0.09
Pulvinar sign	0	0	

White matter lesions were visually assessed on FLAIR images. Pulvinar sign and lacunar lesions were visually rated on T1-weighted images. ^‡^ Statistically significant

**Table 3:** Performance measures for differentiation between MS and FD

	Corpus callosum lesions	Juxtacortical lesions	Infratentorial lesion	Lacunar lesion
Sensitivity	81%	81%	52%	33%
Specificity	95%	86%	86%	95%
Accuracy	64%	63%	58%	56%
Positive predictive value	94%	94%	79%	88%
Negative predictive value	83%	82%	64%	59%
Positive likelihood ratio	17	5.7	3.7	7
Negative predictive value	5	4.5	1.8	1.4

The presence of at least one corpus callosum, juxtacortical and infratentorial lesions defined on FLAIR images was used to calculate performance measures regarding the differentiation of MS from FD, and the presence of lacunar lesion seen of T1-weighted images regarding the differentiation of FD from MS

## Discussion

In this study, we assessed the neuroradiological discrimination of WML in patients with MS from those with vascular lesions due to FD. Our main finding was that the individual differentiation between MS and FD based on the visual assessment of WML distribution on axial FLAIR images had only a moderate interrater reliability despite distinctive group differences.

To our knowledge, no previous study directly investigated blinded interrater reliability of WML on FLAIR images between MS patients and patients with a confirmed genetical disorder causing vascular WML. One study investigated blinded ratings of FLAIR and FLAIR* images by 2 MS specialists in 68 MS patients, 8 healthy subjects, and 11 cases with other neurological diseases and found a high diagnostic accuracy for both sequence modalities for a correct MS diagnosis (area under the receiver operating curve values between 0.9 and 0.95). Interrater reliability was higher than in our study for FLAIR images with a Cohen’s kappa coefficient of 0.68 and the addition of FLAIR* sequences for the detection of a central vein sign (CVS) improved overall diagnostic accuracy slightly further [26]. A possible explanation for the lower accuracy and interrater reliability in our study is the higher number of raters with differences in training background and differences in the study population. Moreover, rating in our study was based on standard FLAIR images alone and did not include additional sequences.

Several studies evaluated the diagnostic performance of imaging criteria between patients with MS and its mimics. One study found that the Barkhof criteria in patients with headache and WML were met in 2.4% when restricted to “touching” lesions and in 7.1% for lesions within 3 mm for periventricular or juxtacortical areas. Likewise the 2010 McDonald dissemination in space criteria were met in 24.4% and 34.5%, respectively [14]. Another study showed that 13.1% of patient with Neuro-Behçet’s disease fulfilled the Barkhof criteria [15] and Kim and colleagues found that the Barkhof criteria could not significantly distinguish WML in patients with MS from those in primary or secondary central nervous system vasculitis and only moderately from WML in systemic lupus erythematodes or Sjogren's syndrome [16]. The difficulty arises in part because WMLs in MS and other disorders might occur partly in similar regions fulfilling the specific spatial prerequisites of the criteria in spite of their characteristic differences in shape or temporal dynamics. Furthermore, the appearance and etiology of WML in SVD is heterogenous [27] and can range from periventricular pencil-thin lining or small punctuated WML as seen in normal aging to more pronounced caps and bands around the lateral ventricles and confluent lesions. Likewise, age-related SVD occurs in patients with MS as well, complicating the radiological differentiation further [28]. Our results underscore these reported difficulties of WML classification at the individual patient level given that 67% of FD patients were incorrectly classified by at least one rater.

A recent study also investigated the application of the McDonald MS diagnostic criteria using an online survey completed by neurological residents and MS specialists. The correct number of periventricular on selected T2-weighted images were identified by only 39% of residents and 52% of MS specialist while the number of juxtacortical lesions were correctly identified by 28% and 53%, respectively [21]. While not directly comparable, our results regarding correct classifications of WML also differed slightly between raters, especially in MS patients. This underscores that at least part of the interrater variability likely depends on the expertise and experience of the raters.

We additionally assessed differences in lesion location to demonstrate that our MS and FD groups differed as expected and therefore were representative for the blinded interrater evaluation. In line with the previous literature, we found that juxtacortical lesions clearly involving the subcortical U-fibers and corpus callosum lesions were highly specific and sensitive for the differentiation between the two patient groups. Corpus callosum involvement especially has been long-known to be a characteristic feature of WML in MS [29–31] and is only rarely found in SVD because of the double vascular supply [1]. Corpus callosum lesions and infratentorial lesions were also found to be highly specific and sensitive in other studies regarding the differentiation of MS from FD [32, 33]. Periventricular or deep white matter lesions were found in all of our patients and the evaluation of the characteristic differences of MS lesions in comparison to those in SVD (e.g. axis perpendicular to the lateral ventricles or having an ovoid shape) have been investigated extensively in the literature [34]. Lacunar lesions were more frequently present in FD patients than in MS but occurred only in about one third of FD patients limiting the sensitivity of this marker. Regarding imaging characteristics of FD, our results further support recent studies, which showed that the pulvinar sign [35] is rarely found in these patients [11, 36]. Apart from imaging characteristics, clinical data differed as expected between patient groups given the frequent affection of multiple organ systems in FD (e.g., cardiomyopathy, renal disease, angiokeratomas or cornea verticillate). Therefore, by combining clinical, laboratory and imaging results as well as positive family history the distinction between MS and FD should normally be feasible in the majority of patients. However, in clinical practice not all information may be available at the time of MRI during initial clinical work-up and it is important to include FD as a potential differential diagnosis of WML detected on MRI to avoid diagnostic delay since treatment options (enzyme replacement therapy or small molecule chaperone therapy in selected patients) are available and symptomatic treatments for cardiac or renal disease help to reduce morbidity and mortality. Furthermore, early diagnosis of FD allows genetic counseling and screening of relatives.

Limitations of this study are the retrospective design with heterogenous non-standardized sequence parameters that could potentially have biased our secondary analyses regarding measures of diagnostic performance. Furthermore, the sample size was small with only 21 patients in each group which might have undermined group comparisons because of low statistical power. Additionally, the range of age differed between the groups which could have impaired data interpretation or diluted group differences because of additional age-related changes on MRI or a correlation of age with different disease stages. Moreover, the MS group was heterogenous regarding the MS subtype and future studies should investigate if interrater reliability differs between RRMS and SPMS/PPMS. Our results also apply to the evaluation of axial FLAIR images only and are not directly comparable to more advanced sequences for the detection of CVS in lesions such as FLAIR* images or 3D-FLAIR sequences. The CVS in particular has been suggested as a specific marker for MS lesions with moderate sensitivity for discrimination against MS mimics [37–41]. However, while a threshold of ≥ 40% CVS + lesions was indicative of MS compared to SVD in one study, only a moderate interrater agreement was found when the 40% CVS cut off was used to establish a correct diagnosis [42], suggesting that further studies are necessary to investigate whether the CVS improves diagnostic accuracy in clinical practice in unselected patients. Moreover, repeated MRI to evaluate dissemination in time as routinely done in clinical practice would likely improve diagnostic accuracy of MS. Finally, we did not assess intrarater reliability which could potentially further impact diagnostic accuracy and should be investigated in future studies.

Summarizing our results, we found that the visual differentiation of WML in MS from vascular WML in FD patients based on axial FLAIR images alone has only a moderate interrater reliability despite the distinctive differences of WML locations. Our findings underscore the importance of specific sequence protocols in the evaluation of WML when a diagnosis of MS is suspected as suggested by recent guidelines to avoid misdiagnosis leading to wrong and potentially harmful treatments [2, 3, 34]. The findings do also underscore the utmost importance to combine clinical, radiological and laboratory findings for accurate diagnoses.

## Data Availability

Data can be made available from the corresponding author upon reasonable request and after approval from the ethics review board at the Medical University of Vienna.

## Abbreviations

CVS: Central vein sign
EDSS: Expanded disability status scale
ERT: Enzyme replacement therapy
FD: Fabry disease
FLAIR: Fluid-attenuated inversion recovery sequence
MRI: Magnetic resonance imaging
MS: Multiple sclerosis
PPMS: Primary progressive multiple sclerosis
RRMS: Relapsing remitting multiple sclerosis
SPMS: Secondary progressive multiple sclerosis
SVD: Small vessel disease
TE: Echo time
TI: Inversion time
TR: Repetition time
WML: White matter lesions
